# EGFR inhibition blocks cancer stem cell clustering and lung metastasis of triple negative breast cancer

**DOI:** 10.7150/thno.57706

**Published:** 2021-04-30

**Authors:** Xia Liu, Valery Adorno-Cruz, Ya-Fang Chang, Yuzhi Jia, Madoka Kawaguchi, Nurmaa K. Dashzeveg, Rokana Taftaf, Erika K. Ramos, Emma J. Schuster, Lamiaa El-Shennawy, Dhwani Patel, Youbin Zhang, Massimo Cristofanilli, Huiping Liu

**Affiliations:** 1 Department of Pharmacology, Northwestern University Feinberg School of Medicine, Chicago, IL, USA.; 2 Department of Toxicology and Cancer Biology, University of Kentucky, Lexington, KY, USA.; 3 Department of Pharmacology, Case Western Reserve University, Cleveland, OH, USA.; 4 The Ben May Department for Cancer Research, the University of Chicago, Chicago, IL, USA.; 5 Laboratory of Functional Biology, Graduate School of Biostudies, Kyoto University, Kyoto, Japan.; 6 Drikskill Graduate Program in Life Sciences, Northwestern University Feinberg School of Medicine, Chicago, IL, USA.; 7 Department of Medicine, the Division of Hematology and Oncology, Northwestern University Feinberg School of Medicine, Chicago, IL, USA.; 8 Lurie Comprehensive Cancer Center, Northwestern University Feinberg School of Medicine, Chicago, IL, USA.; 9 Department of Pathology and Case Comprehensive Cancer Center, Case Western Reserve University, Cleveland, OH, USA.

**Keywords:** cancer stem cells, metastasis, EGFR, circulating tumor cells, CTC cluster, circulating cancer stem cell

## Abstract

Triple-negative breast cancer (TNBC) is one of the most aggressive and metastatic breast cancer subtypes lacking targeted therapy. Our recent work demonstrated that circulating tumor cell (CTC) clusters and polyclonal metastasis of TNBC are driven by aggregation of CD44^+^ cancer stem cells (CSC) and associated with an unfavorable prognosis, such as low overall survival. However, there is no existing therapeutic that can specifically block CTC or CSC cluster formation.

**Methods:** Using patient-derived xenograft (PDX) models, we established an *ex vivo* tumor cell clustering assay for a pilot screening of blockade antibodies. After identifying EGFR as a target candidate, we modulated the gene expression and inhibited its kinase activity to determine its functional importance in tumor cell clustering and therapeutic inhibition of lung metastasis. We also examined the molecular regulation network of EGFR and a potential connection to CSC marker CD44 and microRNAs, which regulate CTC clustering.

**Results:** We report here that EGFR inhibition successfully blocks circulating CSC (cCSC) clustering and lung metastasis of TNBC. EGFR enhances CD44-mediated tumor cell aggregation and CD44 stabilizes EGFR. Importantly, blocking EGFR by a novel anti-EGFR monoclonal antibody (clone LA1) effectively blocked cell aggregation *in vitro* and reduced lung metastasis *in vivo*. Furthermore, our data demonstrated that the tumor suppressor microRNA-30c serves as another negative regulator of cCSC clustering and lung metastasis by targeting CD44 as well as its downstream effector EGFR.

**Conclusion:** Our studies identify a novel anti-EGFR therapeutic strategy to inhibit cCSC aggregation and therefore abolish cCSC cluster-mediated metastasis of TNBC.

## Introduction

Metastasis causes 90% of cancer mortality, and a better understanding its underlying mechanisms is required for better prevention and more effective treatments. In order to metastasize, tumor cells must overcome several barriers, including the initial steps of detachment from the primary tumor, invasion to the surrounding tissue, and intravasation into the peripheral vasculature as well as the late steps of circulation, homing, and colonization at distant organs. Accumulating evidence from our studies and others suggests that cancer stem cells (CSCs) with self-renewal and plasticity are responsible not only for tumor initiation and therapy resistance, but also for mediating distant metastasis 1-12. Within a tumor, such subpopulations of cancer cells with regenerative stemness have the potential for self-renewal, proliferative expansion, lineage plasticity in response to stress and treatment, and aberrant differentiation to heterogeneous progenies 7, 13. More intriguingly, CSCs can possibly escape from immune recognition and attacks from both innate and adaptive immune cells 14-16. Stemness has been demonstrated to be one of the requisites for successful cancer metastasis 4, 10, 12.

Many molecular markers of stemness have been identified in various cancer types, such as CD44 in breast cancer 2 and LGR5 in colon cancer 17-21. Specifically, we found that CD44^+^ breast CSCs are enriched in invasive tumor cell populations 4 and in circulating tumor cell (CTC) clusters that drive polyclonal lung metastases of triple negative breast cancer (TNBC), which lacks expression of estrogen receptor, progesterone receptor, and human epidermal growth factor receptor (HER2) 10. In contrast to the dogma of single cell-mediated dissemination, clustered circulating cancer stem cells (cCSCs) are more tumorigenic and metastatic than single CTCs, with advantageous survival traits and enhanced regenerative power or stemness 10. The presence of CTC clusters is associated with a poor prognosis in breast cancer patients 10. However, CTCs and cCSCs are generally understudied, and there are no existing therapeutics specifically targeting CTCs, CTC clusters, or cCSC clusters. How to target stemness and prevent CTC/cCSC clustering in a therapeutic setting has yet to be discovered for breast cancer treatment.

Our recent studies unveiled a new mechanism of cCSC cluster formation through homotypic cellular aggregation in a CD44-dependent fashion, which is expressed in 80% of CTC clusters 10. We hypothesized that surface molecule-targeting antibodies may interfere and therefore block the cellular aggregation-mediated cluster formation. Using breast cancer patient-derived xenograft (PDX) mouse models that develop CTC clusters and spontaneous metastases to the lungs, we established an *ex vivo* tumor cell clustering assay for a pilot screen of blockade antibodies. We identified a new antibody clone (LA1) against epidermal growth factor receptor (EGFR) that successfully abolished tumor cell cluster formation.

EGFR is a tyrosine kinase frequently overexpressed in TNBC and known to be involved in several cancers by promoting tumor growth and migration 22. Numerous studies have reported that EGFR is a modest to strong prognostic indicator of recurrence-free and overall survival across multiple cancer types, such as breast, head and neck, ovarian, and colorectal cancers 23. However, existing EGFR-inhibitory drugs used to treat head and neck squamous cell carcinoma, lung cancer, and colorectal cancer have a limited response rate in TNBC 23, 24, and the role of EGFR in CTC clusters and polyclonal metastasis has yet to be studied. This study examined the functional importance of EGFR and the molecular mechanisms regulating EGFR in tumor cluster formation, and then evaluated the therapeutic potential of the new anti-EGFR antibody LA1 in blocking metastasis of TNBC *in vivo*.

## Results

### EGFR promotes TNBC cell aggregation/clustering

**Figure 1:**
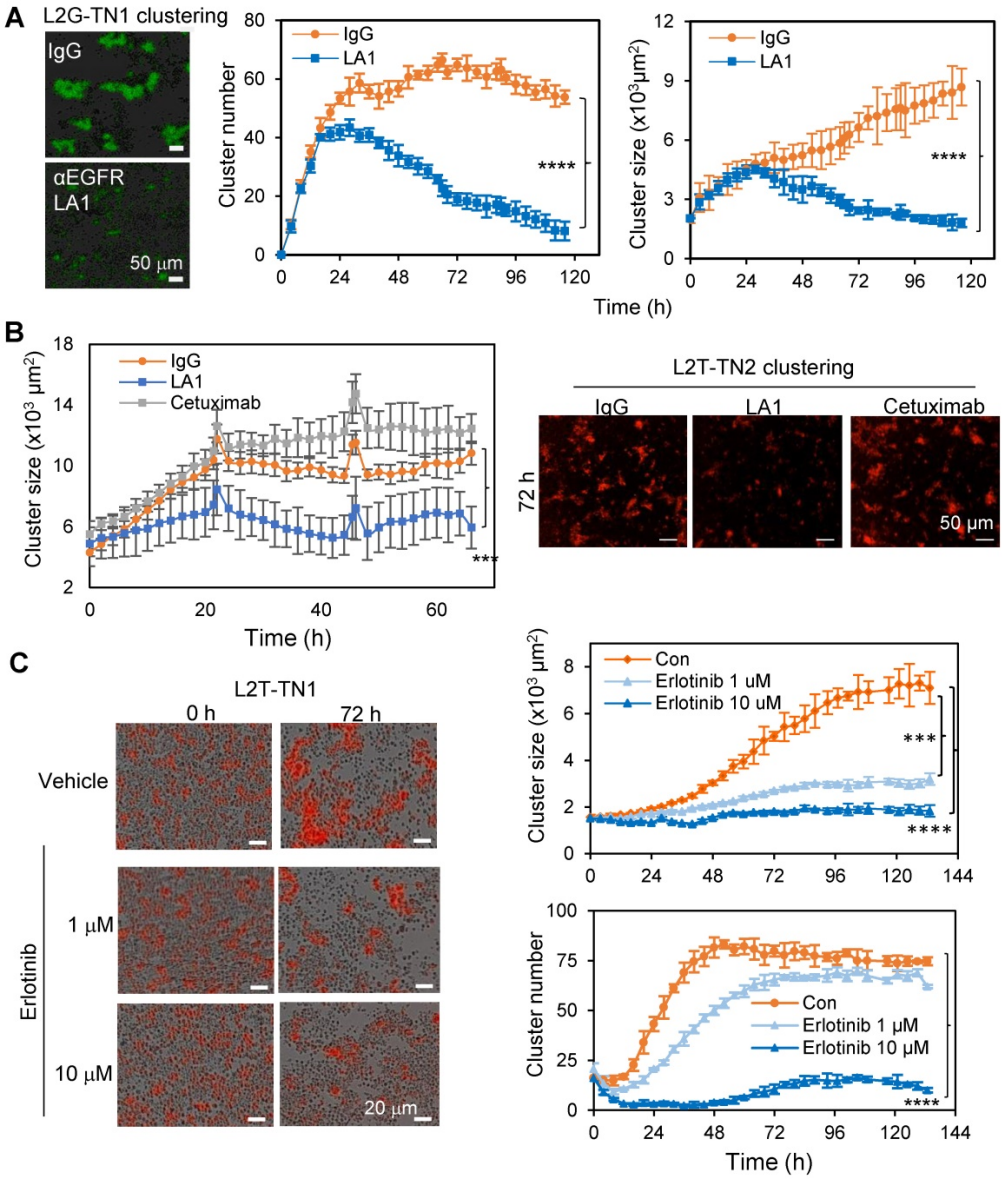
** EGFR inhibition blocks tumor cell cluster formation. A.** Clustering images (left panels) and curve analyses (right panels) of tumor cells from L2G-TN1 PDXs in the presence of IgG or anti-EGFR antibody LA1 during clustering assays (n = 5, ****p < 0.0001 at the end point). **B.** Clustering images (left panels) and curve analyses (right panel) of tumor cells from L2T-TN2 PDXs in the presence of IgG, LA1, and cetuximab during clustering assays. **C.** Clustering images (left panels) and curve analyses (right panels) of tumor cells from L2T-TN1 PDXs in the presence of erlotinib and vehicle (Con) during clustering assays.

We previously established multiple TNBC PDXs which develop spontaneous metastases to the lungs with detectable CTC clusters both in the blood and within the vasculature of tissue sections 4, 10. Using PDX-derived single primary tumor cells in suspension which loosely attached to the collagen I-coated plates, and the IncuCyte-based dynamic imaging approach, we created an *ex vivo* 3D culture system and monitored the tumor cell aggregation and cluster formation in mammary stem cell medium on collagen I-coated plates within 24 to 72 hours as previously described 10. The PDXs were labeled by two optical reporter fusion genes, including Luc2-eGFP (L2G) with the luciferase 2 (Luc2) fused to enhanced green fluorescent protein (eGFP), and Luc2-tdTomato with the Luc2 fused to td-Tomato (L2T) 10. By screening neutralizing antibodies against surface proteins and cytokines, we identified an EGFR monoclonal antibody (mAb), clone LA1, as a strong inhibitor of tumor cluster formation of L2G-labeled TN1 PDX cells (**Figure 1A**). In comparison to the FDA-approved anti-EGFR mAb cetuximab which had no significant effect, LA1 dramatically blocked tumor cell aggregation and cluster formation of two TNBC PDX models, TN1 and TN2, labeled by L2G and L2T respectively (**Figure 1B and Figure S1A**). Additionally, we observed an increased cluster size when the base media was supplemented with EGF (**Figure S1B**). Consistently, administration of the EGFR kinase inhibitor erlotinib (0.1-10 µM) and downstream MEK inhibitor PD 325901 (0.01-1 µM) dramatically reduced PDX-derived tumor cell cluster formation (**Figure 1C and Figure S1C-D**). These data suggest that EGFR activation and its downstream signaling play an essential role in TNBC cell aggregation.

### EGFR enhances CD44-mediated TNBC cell clustering

**Figure 2:**
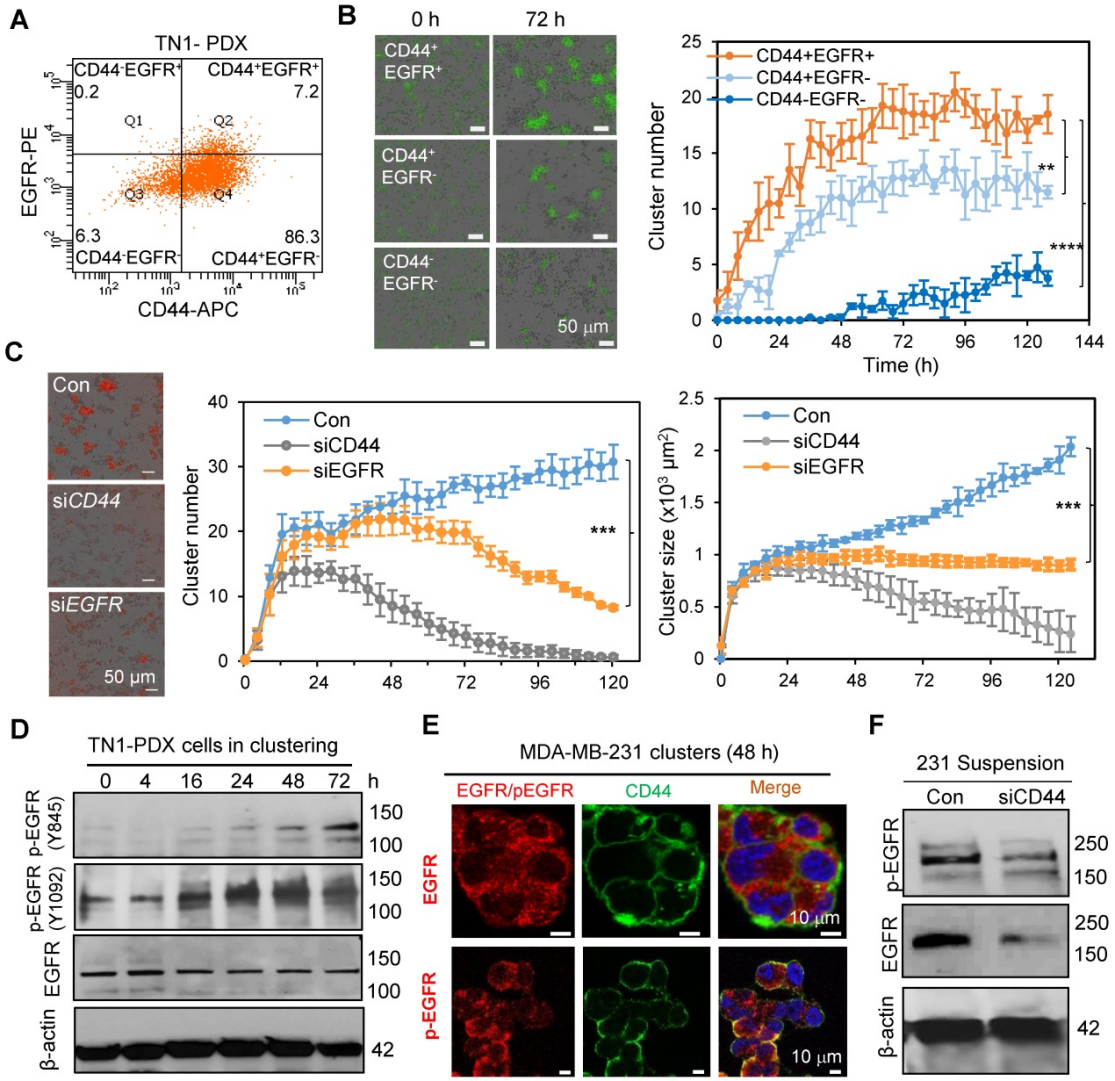
** EGFR enhances CD44 mediated tumor cell clustering. A.** Flow dot plot of CD44 and EGFR expression in TN1 PDX tumor cells. **B.** Clustering images (left panels) and curve analyses (right panel) of tumor cells (CD44^+^EGFR^+^, CD44^+^EGFR^-^, and double negative) sorted from L2G-TN1 PDXs. **C.** Clustering images (left panels) and curve analyses (right panels) of tumor cells from L2T-TN1 PDXs transfected with siRNA control (Con), siCD44, or siEGFR during clustering assays. **D.** Immunoblots of EGFR and phospho-EGFR (Y845 and Y1092) and β-actin (loading control) in TN1 PDX tumor cells at the indicated time points between 0 and 72 h of the clustering assay. **E.** Images of immunofluorescence staining of MDA-MB-231 cells in suspension for EGFR or pEGFR (Y845, red) and CD44 (green) with Dapi (blue)-stained nuclei. **F.** Immunoblots of phospho-EGFR (Y845) and total EGFR in MDA-MB-231 cells in suspension for 48 h after transfection with scramble control (Con) or siCD44.

Our previous work identified the CSC marker CD44 as a driver of TNBC cell aggregation through its homophilic interactions to drive cCSC cluster formation and polyclonal metastasis 10. We first examined whether EGFR coordinates with CD44 in tumor clustering. We evaluated the cluster formation efficiency of sorted PDX tumor cells with different CD44 and EGFR expression. The CD44^+^EGFR^+^ double-positive tumor cells (Q2) showed highest cluster formation ability compared to the CD44^+^EGFR^-^ single-positive cells (Q4) or CD44^-^EGFR^-^ double-negative cells (Q3) (**Figure 2A-B**). Since CD44^-^EGFR^+^ cells (Q1) in TN1 PDX model were relatively rare and not sufficiently sorted for the clustering analysis (**Figure 2A**), we compared the cluster formation abilities of CD44^+^EGFR^+^_,_ CD44^-^EGFR^+^, CD44^+^EGFR^-^ and CD44^-^EGFR^-^ cells sorted from MDA-MB-231 cells (CD44 WT or KO with siEGFR transfection-mediated knockdown) (**Figure S2C**). Consistently, CD44^+^EGFR^+^ displayed the highest cluster formation ability than other three populations (**Figure S2A&B**), suggesting CD44 and EGFR-promoted cluster formation is dependent on each other. We then continued to investigate the functional importance of EGFR in tumor cluster formation by genetic modulation of its expression levels. siRNA-mediated EGFR knockdown resulted in a reduced efficiency of cluster formation (cluster number and size) of TNBC PDX cells, partially mimicking the effects of CD44 knockdown (**Figure 2C**).

Next, we investigated if EGFR is a target of CD44. While EGFR was relatively stable in clustered tumor cells, it was gradually activated during the clustering course of PDX tumor cells (indicated by the phosphorylation of EGFR at Y845 and Y1092, **Figure 2D, Figure S2D**). We also observed that activated EGFR (pY845) was specifically co-localized with CD44 in clustered MDA-MB-231 TNBC tumor cells in suspension, mimicking a suspension condition of CTCs, but not in the adherent cells (**Figure 2E, Figure S2E**), suggesting a specific synergistic interaction between activated EGFR and CD44 during CTC clustering. Upon knockdown of CD44, both total EGFR and activated EGFR (pY845) were significantly decreased in MDA-MB-231 cells in suspension (**Figure 2F**). Consistently, CD44 knockdown also reduced total EGFR and phosphorylation levels of EGFR in human TNBC PDX cells when cultured *ex vivo* to form clusters (**Figure S3A**). However, there was no difference in EGFR mRNA levels upon CD44 knockdown (**Figure S3B**). When protein degradation pathways were blocked by proteasome inhibitor MG-132 and endocytosis inhibitor sucrose, CD44 knockdown-mediated reduction of phosphorlation levels of EGFR were rescued (**Figure S3C**), suggesting that CD44 can prevent EGFR degradation, which is consistent with previous studies 40, 41.

### miR-30c reduces cell clustering and metastasis by targeting CD44 and EGFR

**Figure 3:**
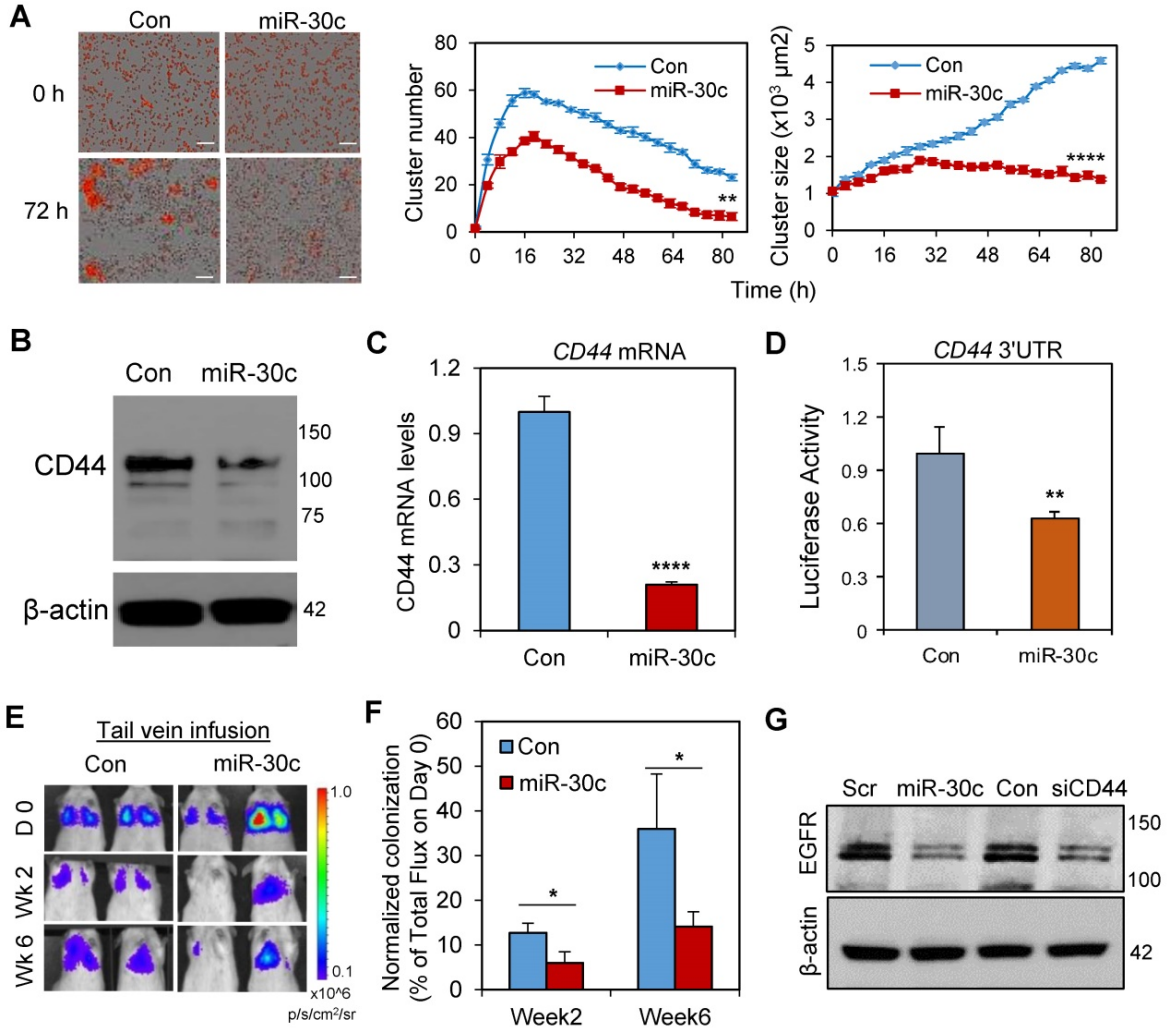
** miR-30c reduces cell clustering and metastasis by targeting CD44 and EGFR. A.** (Left) Representative images of primary TN1 tumor cell clusters upon transfection with miR-30c and the scramble control (Con) at 0 and 72 h time points. Scale bars: 100 μm. (Right) Curves of cluster numbers and cluster size of TN1 cells transfected with miRNA control (cCon) and miR-30c, monitored by IncuCyte time lapse imaging (n = 5, **p < 0.01 and ****p < 0.0001 at the end point). **B.** Immunoblot of CD44 in TN1 breast tumor PDX cells treated with control miRNA (Con) and miR-30c. β-actin was the loading control. **C.** *CD44* mRNA levels in breast tumor cells (TN1) treated with control miRNA or miR-30c (n = 3, ****p = 0.00007). **D.** Luciferase reporter assay of the inhibitory effects of miR-30c on the *CD44* 3'UTR (n = 5, **p = 0.0045). **E.** Bioluminescence images of NSG mice inoculated with 5x10^5^ L2G-TN1 PDX cells (control and miR-30c transfected) via tail vein infusion. The rainbow scale at right indicates photon flux intensity. Colored regions indicate lung metastasis. **F.** Normalized signal of TN1 PDX-mediated lung metastasis (% of day 0 signal) upon tail vein-infusion of tumor cells transfected with miR-30c or scramble control miRNA (Con). **G.** Immunoblot of EGFR in TN1 PDX cells upon miR-30c induction (first two lanes) and CD44 knockdown (last two lanes). Scr, scramble control miRNA.

We previously found that microRNA-30c (miR-30c) is a potential new therapeutic for TNBC, as the restoration or overexpression of miR-30c is effective in reducing CD44^+^ breast CSC-mediated metastasis as well as chemotherapy resistance in PDXs *in vivo* 5, 6. We further investigated if miR-30c regulates tumor cell cluster formation and if miR-30c has any regulatory effects on CD44 and EGFR levels. Not surprisingly, overexpression of miR-30c in PDX-derived TNBC cells resulted in a reduction in both size and counts of PDX tumor clusters *ex vivo* (**Figures 3A**). Furthermore, the expression of miR-30c in CD44^+^ CSCs was lower compared to that of CD44^-^ tumor cells in TNBC PDX models (**Figure S4A**), suggesting a possible negative regulation between CD44 and miR-30c. Indeed, miR-30c overexpression caused a reduction in CD44 mRNA and protein expression (**Figures 3B-C**) as well as other genes related to epithelial-to-mesenchymal transition (EMT) and stemness, such as VIM, SNAI2, TWF1, and BMI1 (**Figure S4B**). A 3'UTR luciferase assay showed inhibitory binding of miR-30c to the 3'UTR region of *CD44*, confirming that *CD44* mRNA is a direct target of miR-30c (**Figure 3D**). When the miR-30c-overexpressing PDX tumor cells were infused into the mouse tail vein, the normalized lung colonization (% of the signal versus that of day 0) was dramatically compromised compared to that of the scramble control group (**Figures 3E-F**). Similar to the effects of CD44 knockdown, miR-30c overexpression also caused a decreased expression of EGFR (**Figure 3G**), suggesting EGFR is a downstream target of the miR-30c-CD44 pathway.

### Inhibition of EGFR reduces experimental lung colonization of TNBC cells

**Figure 4:**
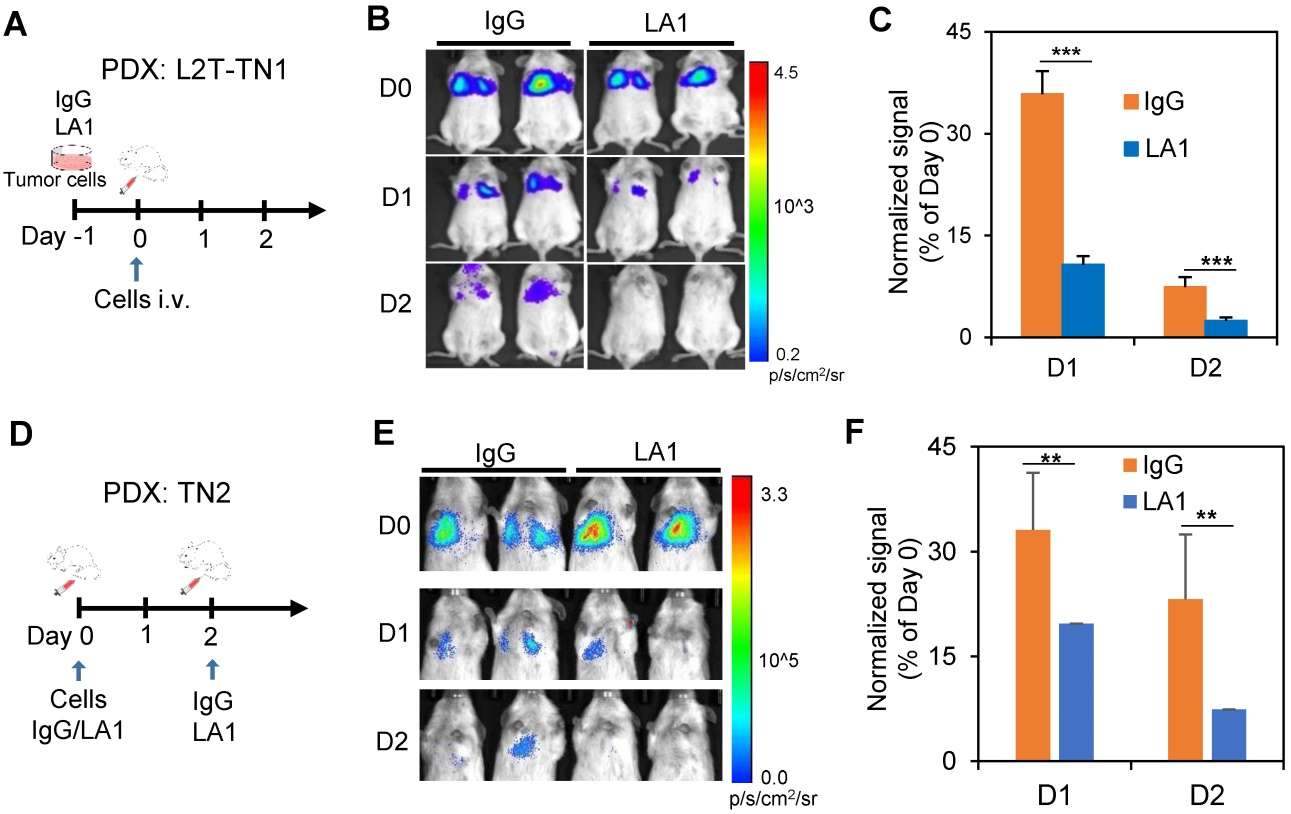
** Inhibition of EGFR effectively blocks lung colonization. A-C.** A schematic of the experimental design for IgG or LA1 treatment *ex vivo*, followed by lung colonization assays (A), bioluminecsence images of mice after tail vein injection of pre-treated TN1 cells on day 0 (D0), 1 (D1), and 2 (D2) (B), and a bar graph showing the normalized changes of lung colonization based on the Day 0 signals (C). **D-F.** A schematic of the experimental design for lung colonization with IgG or LA1 treatment *in vivo* (D), bioluminecsence images of mice after tail vein injection of tumor cells on D0, D1, and D2 (E), and a bar graph showing the normalized changes of lung colonization based on the Day 0 signals after IgG and LA1 treatment *in vivo* (F).

Next, we determined the therapeutic effects of EGFR blockade on experimental metastasis of TN1 and TN2 PDX models *in vivo*. Since LA1 pre-treatment can inhibit tumor cell cluster formation *in vitro* (**Figure 1A&B**), the antibody was either administered to treat the cells before tail vein injections or administered to mice along with or after tail vein injection of cells. Compared to IgG pre-treated cells, the TN1 PDX cells pre-treated with the anti-EGFR antibody LA1 (50 µg/mL) for 16 h had significantly reduced the colonization in the lungs after tail vein injection, measured by bioluminescence imaging (**Figures 4A-C**). In order to determine whether EGFR inhibiton can directly block lung colonization of TN1 PDX cells *in vivo*, LA1 was also directly administered to the recipient mice along with tumor cell injection as well as 2 days after tumor cell infusion. The LA1 anti-EGFR antibody treatment *in vivo* also dramatically reduced lung colonization of TN2 PDX cells (**Figures 4D-F**).

### Inhibition of EGFR blocks spontaneous lung metastasis of TNBC and disrupts CTC clustering

**Figure 5:**
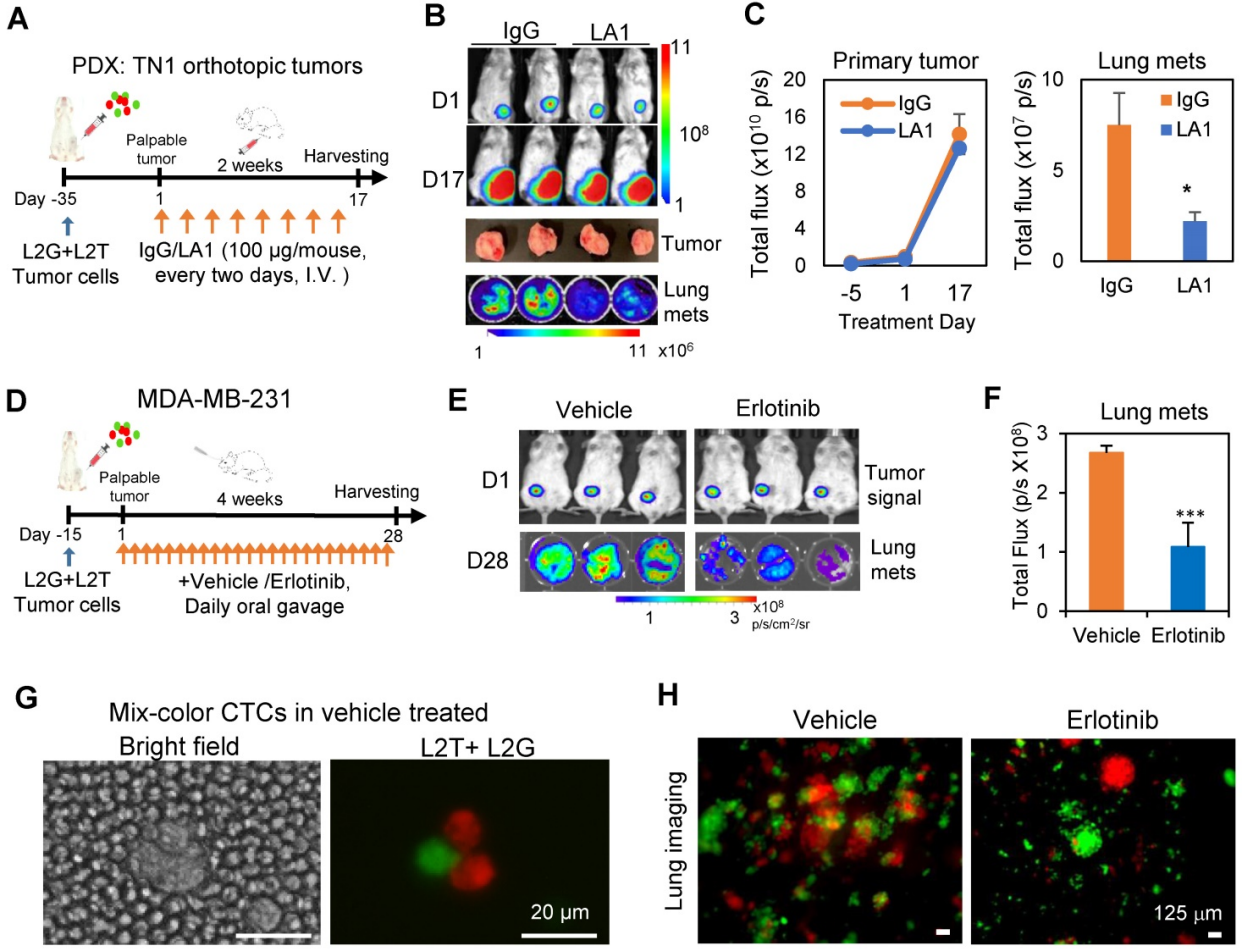
** Inhibition of EGFR blocks spontaneous metastasis. A.** Timeline of tumor inoculation and LA1 treatment after palpable tumor formation in PDX model TN1. **B.** Bioluminescence images of tumor cells orthotopically implanted at the left 4^th^ mammary fat pat on day 1 (D1) , or 17 (D17) of the treatment (top panels), harvested tumors (regular photo, middle panel), and lung metastases (bottom panel). **C.** Tumor growth curve (left panel) and bar graph of lung metastases (mets, right panel) showing comparable IgG and LA1-treated primary tumor signals over time (not significant) but reduced lung mets (one-tailed t-test *p = 0.05). **D.** Timeline of tumor inoculation and erlotinib treatment after palpable tumor formation with orthotopically implanted MDA-MB-231 cells. **E.** Bioluminescence images of tumors on Day 1 (D1) (top panels) and lung metatases (mets) on Day 28 (D28) after treatment with vehicle or 50 mg/kg erlotinib (daily orally) for 4 weeks (bottom panels). **F.** Bioluminescence signal (total flux) histograms of the lungs from vehicle and erlotinib-treated breast tumor-bearing mice as shown in E. **G.** Representative mixed-color tumor cell clusters in blood from tumor-bearing mice treated with vehicle or erlotinib. **H.** Fluorescence images of dissected lungs from tumor-bearing mice treated with vehicle or erlotinib.

To further determine the therapeutic potential of EGFR inhibiton in TNBC, we investigated the effects of EGFR inhibition on spontaneous metastasis. L2G and L2T-labeled PDX tumor cells or MDA-MB-231 cells were orthotopically injected at a 1:1 ratio into the mammary fat pads of NSG mice. Once tumors became nearly palpable, IgG control or LA1 was administered to mice via tail vein infusion (100 µg/mouse, 8 times within 2 weeks). EGFR inhibitor erlotinib was delivered daily via oral gavage for a 28-day treatment. Both LA1 (**Figures 5A-C**) and erlotinib (**Figures 5D-F**) significantly inhibited spontaneous lung metastasis without significant effects on primary tumor growth except for potentially more death regions in the tumor center (**Figure S5A-B**). Interestingly, the percentage of the dual-color polyclonal CTC clusters (L2T^+^L2G^+^ double-positive clusters from aggregated CTCs) within the white blood cells dramatically decreased (about 5-times lower) in the LA1-treated mice compared to that of the IgG treated mice (**Figure S5C**). Consistently, the dual color CTC clusters were only detected in blood from vehicle-treated mice but not in erlotinib-treated mice (**Figure 5G**), and erlotinib treatment dramatically reduced dual color polyclonal metastasis in the lungs (**Figure 5H**).

To further explore the therapeutic potential of LA1 antibody,we then measured possible effects of LA1 on normal mouse blood cells. Consistent with many antibodies used in preclinical and clinical studies, we observed minimal side effects of LA1 on the various populations of blood cells, as part of the toxicity analyses (**Figure S5D**). We also analyzed the EGFR expression and found its levels were enriched in CTC clusters compared to single CTCs isolated from the blood of breast cancer patients (**Figure S6A-B**). Taken together, our data suggest that EGFR inhibition provides a novel approach of disrupting CTC cluster formation and blocking TNBC metastasis.

## Discussion

TNBC lacks expression of estrogen receptor, progesterone receptor, and HER2. It represents about 15-20% of breast cancers and is one of the most aggressive and metastatic breast cancer subtypes. Since it does not respond to endocrine and anti-HER2 therapy, TNBC lacks targeted therapy. In this study, our data revealed a new role of EGFR in metastasis in the promotion of CD44-mediated cell aggregation. Since the presence of CD44^+^ CTC clusters is correlated with a poor prognosis in patients with breast cancer 10, we anticipate better therapeutic effects of EGFR inhibition in patients with CD44^+^EGFR^+^ CTC clusters, which might serve as a new biomarker to predict the TNBC patient's response to EGFR-targeted treatment. Furthermore, we discovered a novel anti-EGFR monoclonal antibody LA1, that effectively inhibits CTC clusters and TNBC metastasis *in vivo*.

EGFR belongs to the ErbB family of receptor tyrosine kinases and is frequently mutated and/or overexpressed in different types of human cancers, including breast, lung, colorectal, and head and neck 23, 25. Several EGFR-targeted therapeutics have been developed and approved to treat cancer patients, such as the tyrosine kinase inhibitor (TKI) erlotinib and anti-EGFR antibody cetuximab 22, 26, 27. Approximately 80% of TNBC overexpresses EGFR 28, making it an attractive and promising therapeutic target in this subtype. However, anti-EGFR treatments have shown limited response rates in breast cancer 29-31, possibly due to the heavy pretreatment mixed subgroups of unselected breast cancer patients. Indeed, in one clinical trial in selected metastatic TNBC patients, the combination of cetuximab with cisplatin doubled the overall response rate, and prolong progression-free survival and overall survival 32. Although a study reported that 11% of TNBC patients harbor EGFR gene mutations 33, other studies indicated that activating mutations in EGFR gene are rare in TNBC 34-36, which may explain the low afficacy of EGFR TKI in unselected breast cancer patients. Therefore, many factors have to be considered to stratify breast cancer patients who are likely to benefit from EGFR-targeted therapies in future clinical trials. It is worth noting that the monoclonal antibody LA1 but not cetuximab can inhibit cancer cell clustering. The LA1 neutralizing antibody binds to the distal half of the EGFR extracellular domain (carboxyl-terminal half of subdomain III or subdomain IV) where the ligand EGF binds 37, 38. Incubation with LA1 inhibits EGF-induced activation of pEGFR, pMAPK and pSTAT3 38. The function of LA1 is consistent with the effects of erlotinib and MEK inhibition on tumor cell cluster formation whereas cetuximab lacks inhibition in low EGFR-expressing breast cancer cells 39, 40 with a very low response rate in the randomized phase II clinical trial 41. Another explanation could be the binding site of LA1 interferes with the synergistic interaction or colocalization of EGFR with CD44, and therefore influences CD44 homophilic interaction-mediated cluster formation and metastasis 10, 42.

CTCs seed metastasis; however, it is a challenging job to target this rare population (<0.1%) in the blood. Our study and others have shown that CTC clusters have 20-100 times higher metastatic potential compared to single CTCs 10, 43. Therefore, understanding the biology of CTC clusters and the pathways regulating their formation are critical to developing strategies to overcome the pathways responsible for their enhanced metastatic ability. Our previous studies have found that breast CSC marker CD44 serves as a therapeutic target 10. CD44 drives tumor cell aggregation via its homophilic interactions, and depletion of CD44 can inhibit cell aggregation and metastasis of TNBC 10. In this study, we further demonstrated that miR-30c overexpression targets both CD44 and EGFR, thereby inhibiting cell aggregation and metastasis. Future CTC cluster blocking strategies can combine multi-modality strategies targeting both CD44 and EGFR with a potential microRNA delivery system or potent anti-CD44 antibodies. Similarly, other studies from the Aceto group demonstrated the stemness of CTC clusters and inhibition of the Na^+^/K^+^ ATPase using ouabain leads to CTC cluster dissociation and remarkable reduction in spontaneous metastasis formation in xenograft models 44. These findings provide a rationale for targeting CTC clusters and inhibiting stemness of CTC clusters to inhibit and prevent metastasis.

We found that CD44 sustains EGFR protein levels by promoting the stability and activity of phosphorylated EGFR during CTC cluster formation. This is consistent with previous studies showing that CD44 inhibits Rab7A-mediated EGFR degradation when cells are under attachment conditions 45, 46. Our data further suggest that CD44 regulates EGFR stability when cells are detached or in circulation. Consistently, ErbB2-positive cells can stabilize EGFR by multicellular aggregation during extracellular matrix detachment 47, 48. Interestingly, a recent study found that stabilizing EGFR protein promotes breast cancer stemness 49. Other studies have also shown that EGFR plays critical roles in the survival, maintenance, and function of CSCs 50-52. It is known that CTC clustering enhances stemness of CTCs 10, 44. Therefore, it is likely that blocking EGFR can inhibit stemness of CTC clusters to reduce metastasis. Consequently, CTC clusters may be uniquely sensitive to EGFR-targeted therapy.

It is likely that early treatment is necessary for prevention of metastasis. However, anti-tumor-specific EGFR and CD44 treatments will also activate immune cells for secondary killing in addition to direct inhibition of tumor aggregation or clustering. We anticipate that these therapeutic targeting strategies will be beneficial to patients with established metastasis. Another strategy is to combine the CTC cluster blockade antibodies with immune checkpoint inhibitors such as anti-PD-L1 or anti-PD1 antibodies. Overall, the future possibilities for treating metastatic cancer continue to advance for precision and personalized medicine as we continue to investigate the mechanisms and the heterogeneity of metastasis.

## Materials and Methods

### Human studies and animal work

All human blood specimen analyses complied with NIH guidelines for human subject studies and were approved by the Institutional Review Board at Northwestern University. The investigators obtained written informed consent from all subjects whose blood specimens were analyzed. All animal procedures complied with the NIH Guidelines for the Care and Use of Laboratory Animals and were approved by Northwestern University Institutional Animal Care and Use Committees (IACUC). Animals were randomized by age and weight. All mice used in this study were kept in specific pathogen-free facilities in the Animal Resources Center at Northwestern University. The criteria for excluding mice from experiments or data analyses were sickness or conditions unrelated to tumors, such as infections of immuno-compromised mice. Sample sizes were determined based on the results of preliminary experiments.

### Patient CTC analysis

Blood samples from breast cancer patients were collected in EDTA tubles for flow cytometry analyses of live cells or collected in CellSave preservative tubes for CellSearch platform analyses. Live cells were centrifuged and resuspended in red blood cell lysis buffer to remove red blood cells. The remaining cells were washed and stained with antibodies for different makers such as EpCAM for epithelial cells, CD45 for leukocytes, and candidate markers such as EGFR, and then analyzed through the FACS LSR (BD Biosciences) instrument. Single and clustered EpCAM^+^ tumor cells were gated for EGFR expression (%). Two sample paired *t*-test were used to compare the proportion of EGFR^+^ tumor cells within the singlets and clustered CTCs. CellSearch kit and anti-EGFR antibody (conjugated to PE, BD Bioscience cat# 555997) were used to enrich CTCs for immunofluorescence staining.

### PDXs, mouse models and tumor dissociation

Multiple TNBC PDXs were established as previously described 4. 8-10 week NOD/SCID or NSG mice were used for PDXs and human MDA-MB-231 cell-based xenograft studies. PDXs and MDA-MB-231 cells were lentivirally labeled by eGFP, tdTomato, Luc2-eGFP (L2G), or Luc2-tdTomato (L2T) using the lentiviruses and labeling protocol described previously 4. PDX tumors were harvested and dissociated either with collagenase III (TN1 model) or Liberase TH and TM research grade (TN2 model and lung tissues). Briefly, tumors were minced and incubated for 2-4 h at 37 °C with collagenase III (Worthington Biochemical) or Liberase TH and TM (Roche) and 100 Kunitz U of DNase I (Sigma) in 20 mL of RPMI medium with 20 mM Hepes buffer. Single-cell suspensions were filtered through 40-μm nylon cell strainers and washed with Hanks' balanced saline solution (HBSS; Sigma) containing 2% heat-inactivated fetal bovine serum (FBS). Red blood cells were lysed with ACK lysis buffer, and dissociated bulk tumor cells were either cultured or stained with various antibodies in HBSS/2% FBS for further flow analysis or sorting on a BD FacsAria cytometer (BD Biosciences). DAPI and H2K^d^ were used as markers for viability and mouse stromal cells, respectively.

### Cell culture and transfections

MDA-MB-231 cells were purchased commercially from ATCC, and verified to be mycoplasma-negative using Lonza's MycoAlert Mycoplasma Detection Kit. Cells were maintained in DMEM with 10% FBS + 1% penicillin-streptomycin (P/S). Primary tumor cells were cultured in HuMEC ready medium (Life Technologies) plus 5% FBS and 0.5% P/S, on collagen type I (BD Biosciences) coated plates. MicroRNAs (Dharmacon, negative control #4) and siRNAs (pooled) (Dharmacon, negative control A) were transfected using Dharmafect (Dharmacon) at 100 nM.

### EGFR inhibition treatment *in vivo*

The LA1 antibody is mouse anti-human EGFR monoclonal antibody and obtained from Millipore (Millipore, cat#05-101-KL). For lung colonization assays, 5×10^5^ PDX cells (TN1 or TN2) were injected *via* the tail vein into NSG mice. Mice were pre-treated two hours before cell infusion. For spontaneous metastasis studies, 5×10^5^ TN1 cells were orthotopically implanted to the mammary fat pads of NSG mice and treated with the EGFR monoclonal antibody LA1 or IgG control once palpable tumors formed. The antibody or IgG (100 µg per mouse) was injected *via* the tail vein every two days.

For erlotinib treatment, 5×10^5^ L2G and 5×10^5^ L2T labelled MDA-MB-231 cells were orthotopically co-injected into NOD/SCID mouse mammary fat pats. After palpable tumor grew (4 weeks), animals were randomized into two groups and the treatment started. Erlotinib (OSI-744, Selleckchem, 100 mg/kg) was administered orally via gavage in 0.5% methylcellulose, 0.2% Tween 80 in sterilized water once a day. Vehicle group received oral gavage of 0.5% methylcellulose, 0.2% Tween 80 in sterilized water. After treatment for 4-5 weeks, mice were euthanized and lungs were imaged by BLI and fluorescence microscopy.

### Co-immunoprecipitation for CD44 and EGFR interaction analysis

Two cDNA plasmids, pCMV6-Flag-CD44 (OriGene, RC221820) and LNCX-EGFR-GFP, were transfected into HEK-293 cells using transfection agent PolyJet (SigmaGenLaboratories, SL100688). At 48 h post transfection, cells were trypsinzed and the dissociated cells in suspension mixed on the Poly-HEMA (Sigma-Aldrich, P3932-10G) coated dish for 3-6 h to make clusters. Adherent cells we collected directly from the dishes by scratching.

After two washes in cold PBS, the cell pellet was frozen for 2 h at -20 °C and then lysed in lysis buffer (250 mM Sucrose, 10 mM Tris, 2 mM EDTA, pH 7.4) with protease inhibitor cocktail (Thermo Fisher Scientific, 78440) and homogenized by 27-gauge needle on ice. After a short spin at 1,000 g for 10 m at 4 °C, the supernatants were further ultracentrifuged at 60,000 g at 4 °C for 30 min to enrich the membrane proteins. The membrane pellet of proteins was resuspended in IP lysis buffer (Thermo Fisher Scientific, 87787) with protease inhibitor cocktail and incubated for 30 min on ice. Protein concentration was determined by Protein Assay Dye Reagent (Bio-RAD, 500-0006). For Co-immunoprecipitation, equal amounts of protein were incubated with anti-Flag M2 Magnetic Beads (Sigma-Aldrich, M8823-5ML) overnight at 4 °C. To elute the protein complex, after 5-10 m incubation with 0.1 M glycine (pH 2-3), the supernatant was collected and the same amount of Tris-Buffered Saline (TBS) was added for neutralizing. For western blotting, samples were diluted in 10% β-mercaptoethanol contained SDS-PAGE loading buffer (Bio-RAD 4Laemmli Sample Buffer Cat. #161-0747). Proteins were resolved by size using 7.5% SDS-PAGE gel and transferred to 0.2 μm pore size of Nitrocellulose membranes. The 5% BSA in TBS/0.1% Tween 20 was used to block the membrane before the incubation with primary antibodies, anti-Flag (1:1000, Sigma-Aldrich, F1804-200UG) or anti-EGFR (1:1000, R&D Systems, AF231), for 1 h at room temperature or 4 °C overnight. After two washes with TBS/0.1% Tween 20, the membrane was incubated with Horseradish peroxidase (HRP)-conjugated secondary antibodies (mouse: 1:10000, Promega, 402B, goat: 1:5000, Santa Cruz Biotechnology, sc-2020) for 1 h at room temperature. HRP signals were detected by enhanced chemiluminescence (ECL) (Thermo Fisher Scientific, 32132).

### Western blots for other proteins

Cells were washed twice in cold PBS, and then lysed by RIPA buffer (50 mM Tris-HCl pH7.4, 1% NP-40, 0.25% sodium deoxycholate, 150 mM NaCl, 1 mM EDTA, 1 mM NaF, 2 mM Na3VO4, 1 mM PMSF) supplemented with Amresco protease inhibitor cocktail (1:100 diluted). The lysate were centrifuged for 10 min at 4 ºC and 1,000 g to remove the debris. Protein concentration in the supernatant was measured and 20 µg of protein were loaded for each sample to SDS-PAGE gels. PVDF membranes were used for protein transfer and blocked with 2% BSA/PBS for 1h at the room temperature (RT), and then incubated with primary antibodies for 1 h at room temperature or overnight at 4 °C, and horseradish peroxidase(HRP)-conjugated secondary antibodies for 1h at RT. The primary antibodies include EGFR (Santa Cruz Biotechnology cat# sc-03), p-EGFR (Santa Cruz Biotechnology cat# sc-23420), and β-actin (Abcam cat# A5441).

### RNA extraction and real-time PCR

Total RNAs were extracted using Trizol (Invitrogen), and RNA was precipitated with isopropanol and glycogen (Invitrogen). After reverse transcription reactions, real-time PCR for miRNAs/genes was performed using individual microRNA/gene Taqman primers (Applied Biosystems) on an ABI 7500 real time PCR system. RNU44 and U6 primers were used for miRNA internal controls and GAPDH for a housekeeping gene control.

### Bioluminescence imaging

The bioluminescence signals of L2G- or L2T-expressing tumor cells were imaged when the substrate of luciferase, luciferin, was added to the medium or injected to mice. Mice bearing these tumor cells were injected intraperitoneally (i.p.) with 100 μL of D-luciferin (30 mg/mL, Gold Biotechnology). After 5-10 min, mice were anesthetized with isoflurane, and bioluminescence images were acquired using the Xenogen IVIS spectrum system (Caliper Life Sciences). Acquisition times ranged from 1 s to 5 min. Signals of the region of interest are analyzed using Living Image 3.0 software (Caliper Life Sciences) and presented as normalized fold change or percentage of the total flux (photons/s, p/s).

### Cell clustering assay

Freshly dissociated primary tumor cells in single cell suspension were seeded in collagen type I-coated 96 well plates. The plates were put into the IncuCyte live cell imaging system (Essen BioScience), and live images were taken every 2-4 h for up to one week. The cluster number and size (µm^2^) were analyzed by Incucyte ZOOM software (Essen BioScience). In the figure panels using cluster size (µm^2^) as Y axis, based on the area of 200 µm^2^ per tumor cell, the approximate cell numbers in a cluster can be calculated using cluster area divided by 200 µm^2^. The mean values and standard deviation (S.D.) were calculated from 3-5 wells (2 images per well of 96-well plates) of each group. In specific experiments, primary tumor cells might be sorted based on the expression of CD44 and EGFR prior to seeding. In other experiments, seeded tumor cells might be transfected with siRNAs (100 nM) or treated with various inhibitors during the clustering assays. For cell viability analysis during clustering, the IncuCyte® Cytotox Red reagent (Essen BioScience) was added into the medium according to the manufacturer's instructions.

### Statistical analysis

Student's t-test was performed for the statistical analyses between 2 samples as appropriate. Using GraphPad Prism 8.1.1 software, one way ANOVA (followed by Tukey posttest) was performed to analyze differences among multiple groups. Data are presented as mean ± SD from at least three replicates. Two sample paired *t*-test were used to compare the proportion of EGFR^+^ tumor cells within the singlets and clustered CTCs. Probabilities under 0.05 are considered significant and indicated with one asterisk (*). Probabilities under 0.01 are indicated with two asterisks (**), under 0.001 with three asterisks (***), and under 0.0001 with four asterisks (****).

## Supplementary Material

Supplementary figures.

## Abbreviations

CTC: circulating tumor cell
EGFR: epidermal growth factor receptor
FBS: fetal bovine serum
HER2: human epidermal growth factor receptor
L2G: Luc2-eGFP
L2T: Luc2-tdTomato
miR-30c: microRNA-30c
mAb: monoclonal antibody
PDX: patient-derived xenograft
P/S: penicillin-streptomycin
TNBC: triple-negative breast cancer
