# Supplementary material for: EGFR inhibition blocks cancer stem cell clustering and lung metastasis of triple negative breast cancer
Source: Theranostics. 2021 Apr 30;11(13):6632–43. doi: 10.7150/thno.57706 (PMC8120216; doi:10.7150/thno.57706)
Supplement: Supplementary file 1 — Supplementary figures. [file thnov11p6632s1.pdf]

Supplementary Figures S1-6

EGFR inhibition blocks cancer stem cell clustering and lung metastasis of triple negative breast cancer

Xia Liu[1,2*, #], Valery Adorno-Cruz[1,3*], Ya-Fang Chang[4], Yuzhi Jia[1], Madoka Kawaguchi[5], Nurmaa K. Dashzeveg[1], Rokana Taftaf[1,6], Erika K. Ramos[1,6], Emma J. Schuster[1,6], Lamiaa El-shennawy[1], Dhwani Patel[1], Youbin Zhang[7], Massimo Cristofanilli[7], Huiping Liu[1,7,8,9, #]

[1]Department of Pharmacology, Northwestern University Feinberg School of Medicine, Chicago, IL, USA. [2] Department of Toxicology and Cancer Biology, University of Kentucky, Lexington, KY, USA. [3]Department of Pharmacology, Case Western Reserve University, Cleveland, OH, USA. [4]The Ben May Department for Cancer Research, the University of Chicago, Chicago, IL, USA. [5]Laboratory of Functional Biology, Graduate School of Biostudies, Kyoto University, Kyoto, Japan. [6]Drikskill Graduate Program in Life Sciences, Northwestern University Feinberg School of Medicine, Chicago, IL, USA. [7]Department of Medicine, the Division of Hematology and Oncology, Northwestern University Feinberg School of Medicine, Chicago, IL, USA. [8]Lurie Comprehensive Cancer Center, Northwestern University Feinberg School of Medicine, Chicago, IL, USA. [9]Department of Pathology and Case Comprehensive Cancer Center, Case Western Reserve University, Cleveland, OH, USA.

*These authors contributed equally to the work.

[#]**Corresponding author**: Huiping Liu, MD, PhD, Northwestern University, 303 E Superior St, Chicago, IL 60611. Email: huiping.liu@northwestern.edu. Xia Liu, PhD, University of Kentucky, 760 Press Avenue, Lexington, KY 40536. Email: xia.liu@uky.edu

Figure S1. PDX tumor cell clustering is blocked by anti-EGFR mAb LA1

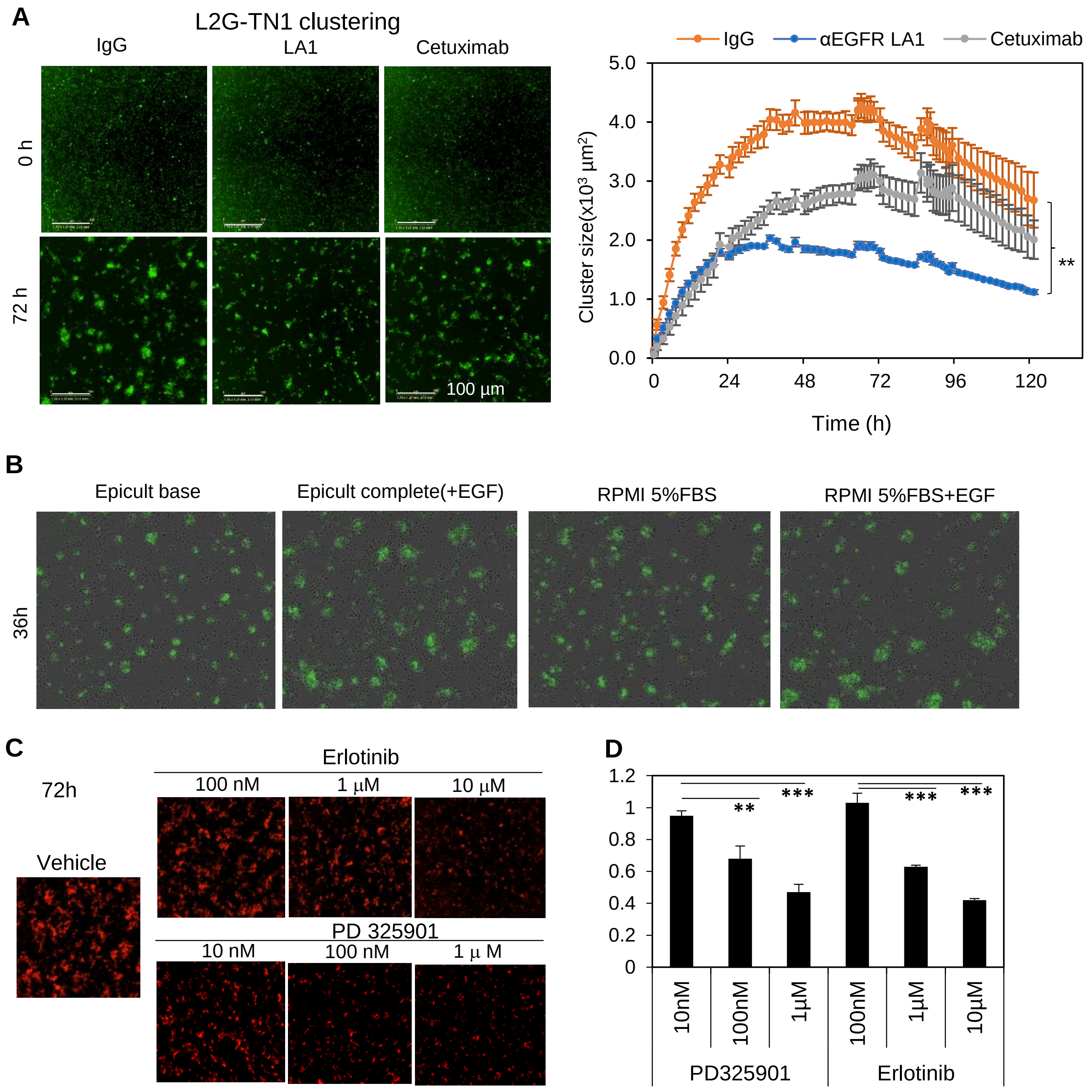

Figure S1. PDX tumor cell clustering is blocked by anti-EGFR mAb LA1

A. Clustering assay of L2G-TN1 cells treated with anti-EGFR mAb LA1 vs cetuximab.

B. Cell cluster formation of TN1 primary tumors in collagen I-coated plates in various culture media: Epicult Base (B base medium only), Epicult complete with EGF (Epicult B base medium plus 5% FBS, supplements with EGF, and 0.48 g/mL hydrocortisone), RPMI 5%FBS (RPMI with 5% FBS and 0.48 g/ml hydrocortisone); RPMI 5% FBS+EGF (with 5% FBS, 0.48 g/ml hydrocortisone, and 10 ng/mL EGF).

C-D: Representative images (C) and quantitative analyses (D) of reduced cell clusters of TN1 PDX tumor cells treated with EGFR inhibitor Erlotinib and MEK inhibitor PD 325901 in a dose-dependent manner. n = 5, $^{**}p < 0.01$, $^{***}p < 0.001$.

Figure S2. EGFR enhances CD44 mediated tumor cell clustering.

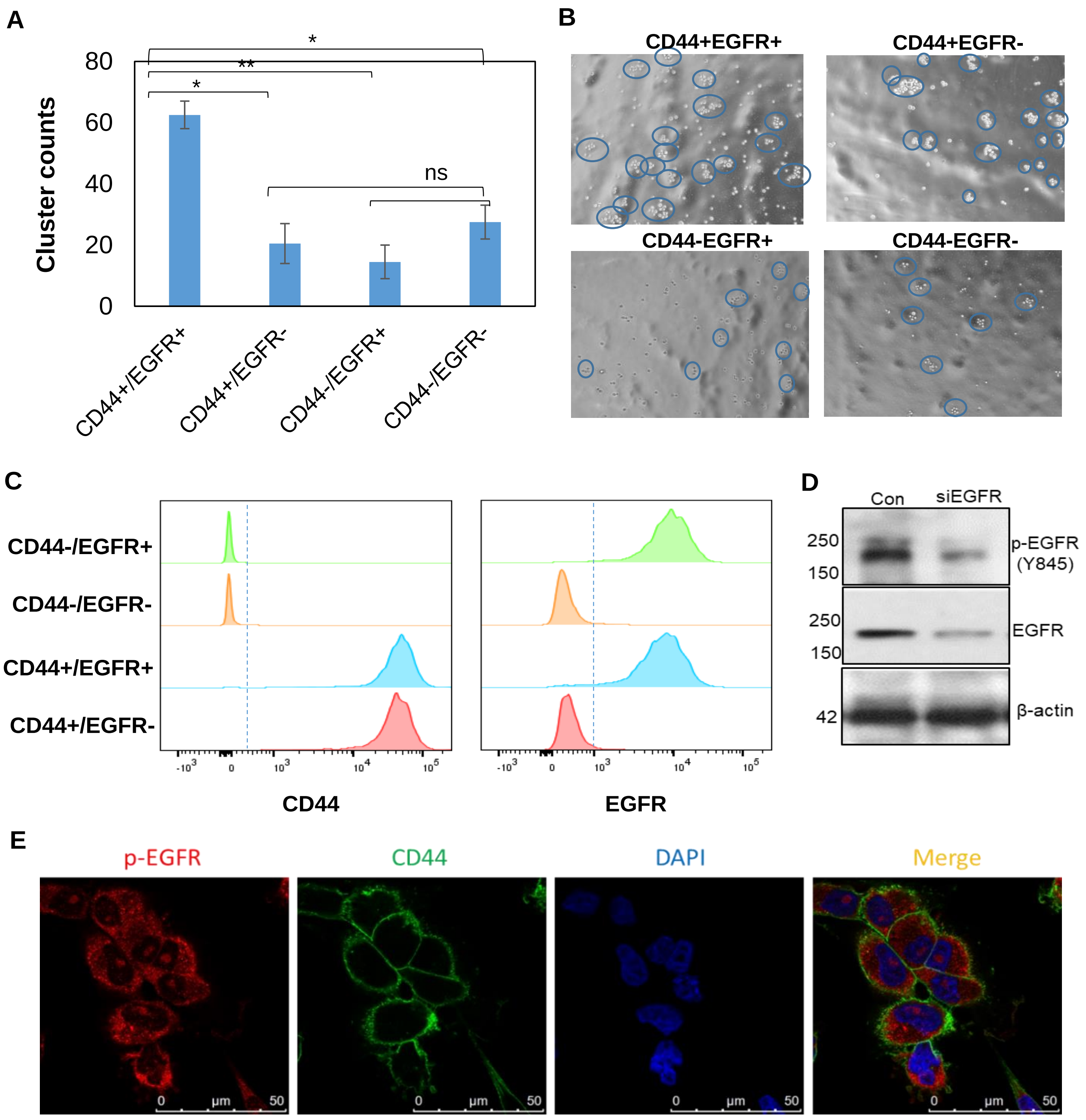

Figure S2. Clustering formation is impaired upon loss of CD44 or EGFR expression.

A. Cluster counts of MDA-MB-231 cells with double positive, single positive, or double negative for CD44 and EGFR expression; n = 2; student t-test one tailed (*p < 0.05, **p < 0.01).
B. Representative images of cluster counts of four CD44/EGFR populations.
C. Flow cytometry validation of CD44/EGFR positive or negative expressions in all 4 populations.
D. Immunoblots of pEGFR (Y845) and EGFR upon knockdown of *EGFR* in TN PDX cells.
E. Images of immunofluorescence staining of adherent MDA-MB-231 for pEGFR (Y845, red) and CD44 (green) with Dapi (blue)-stained nuclei. Scale bars = 50 μm.

Figure S3. CD44 promotes EGFR stability and activity in clusters.

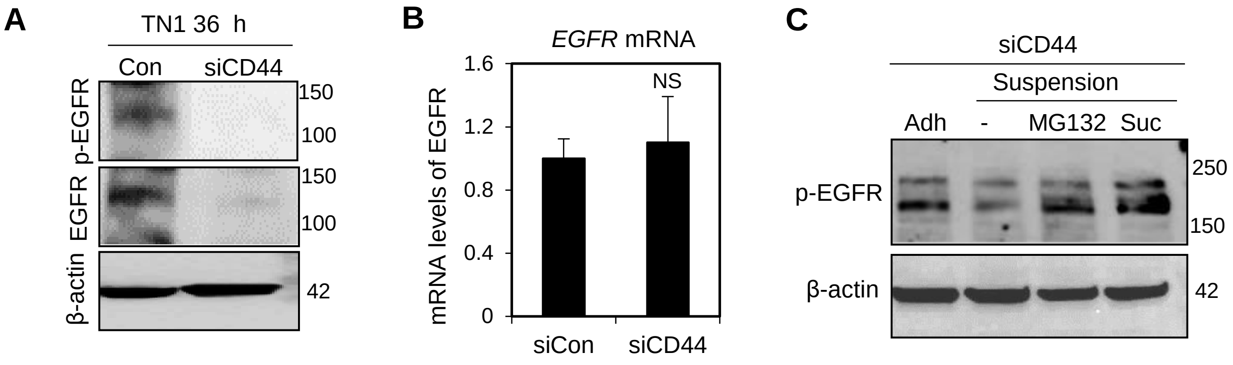

Figure S3. CD44 promotes EGFR stability and activity in clusters.

A. Immunoblots of phospho-EGFR (Y845), EGFR, and actin in TN1-PDX tumor cells in clustering assays after CD44 knockdown for 36 h.
B. Real-time PCR of EGFR mRNA levels after siCD44 knockdown (NS = no significant changes).
C. Immunoblots of phospho-EGFR (Y845) in MDA-MB-231 cells, transfected with siCD44, either adherent (adh) or in suspension in the presence of proteasome inhibitor (MG-132) or endocytosis inhibitor (sucrose) for 6 h.

Figure S4. miR-30c levels in CD44 high and low breast cancer PDXs.

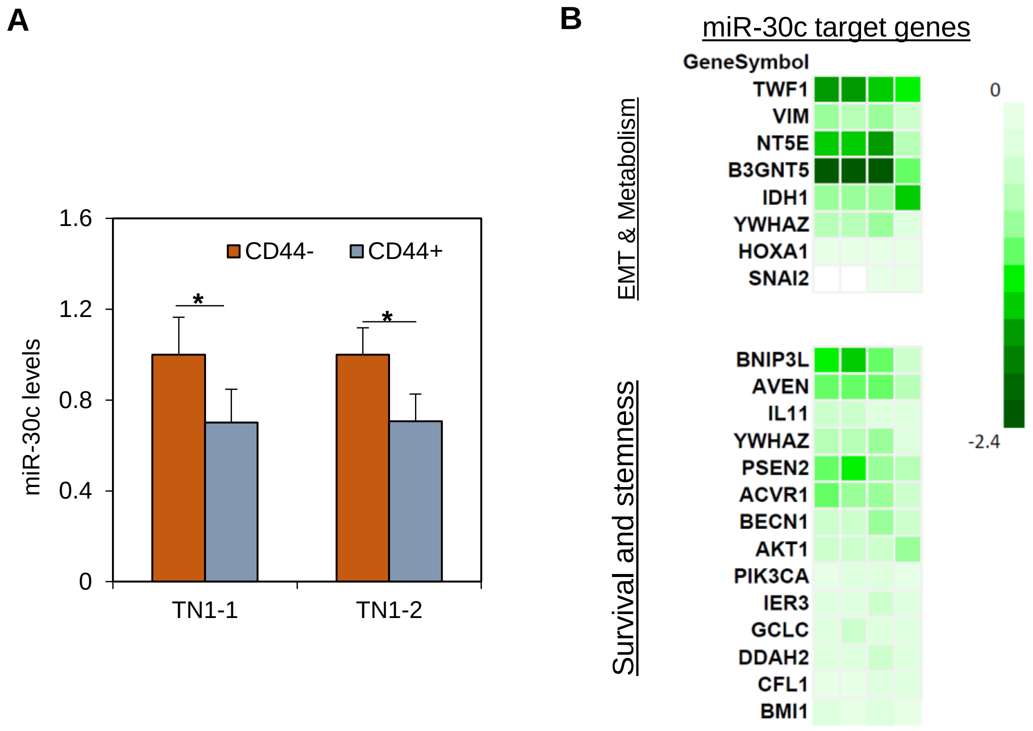

Figure S4. miR 30c levels in CD44 high and low breast cancer PDXs.

A. Real Time PCR of decreased miR-30c expression in $CD44^+$ versus $CD44^-$ tumor cells of both TN1 and TN2 models (n = 3, *p = 0.03 and 0.01 for TN1 from mouse 1, TN1-1, and mouse mouse 2, TN1-2, respectively).

B. **B.** Green box heatmap for the genes inhibited by miR-30c, related to EMT, metabolism, stemness and survival, are color-coded based on log2-transformed expression reduction levels for four pairs of 30c/scrambled microarray comparisons. inhibited by for miR-30c.

Figure S5. Reduced dual-color CTC clusters in LA1-treated mice and minimal toxicity.

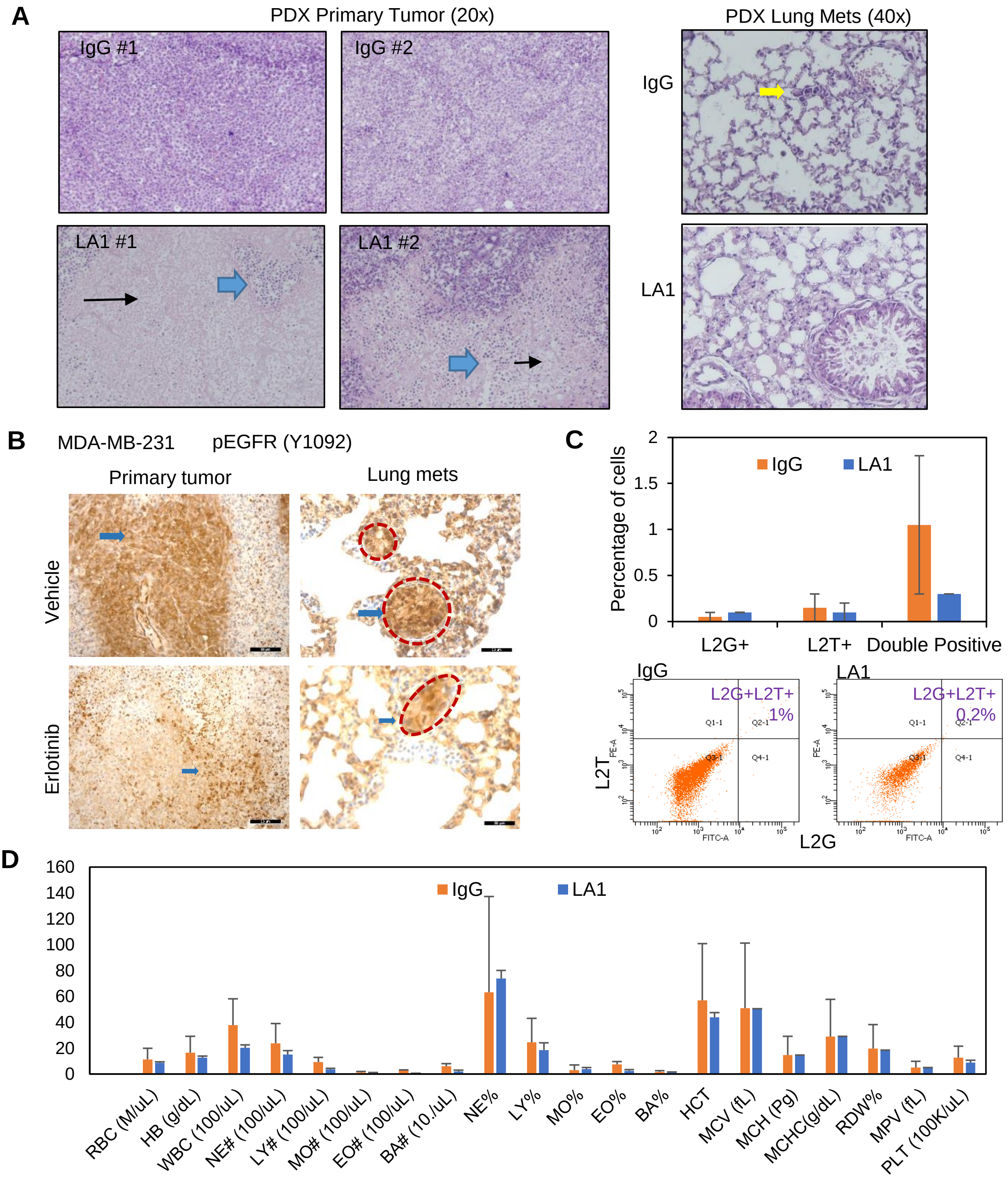

Figure S5. Reduced dual-color CTC clusters in LA1-treated mice and minimal toxicity.

A. Images of H&E staining show LA1 caused death (black arrows) and immune cell infiltration (blue arrows) to the central regions of PDX primary tumors, and abolished lung micromets (yellow arrow in the IgG treated lung and absent in the LA1-treated group).
B. IHC images of pEGFR (Y1092) levels (blue arrows) in IgG/LA1-treated primary tumors and lung mets.
C. Decreased dual-color ($L2G^{+}L2T^{+}$) CTC clusters in LA1-treated mice-bearing L2G/L2T dual-color PDXs.
D. Blood cell counts of both IgG- and LA1-treated mice. RBC: red blood cells, HB: hemoglobin, WBC: white blood cells, NE: neutrophils, LY: lymphocytes, MO: monocytes, EO: eosinophils, BA: basophils, HCT: hematocrit, MCV: mean cell volume, MCH: mean cell hemoglobin, MCHC: mean cell hemoglobin concentration, RDW: red cell distribution width, MPV: mean platelet volume, PLT: platelet count.

Figure S6. Clustered cells with increased EGFR expression compared to single cells in the blood of breast cancer patients.

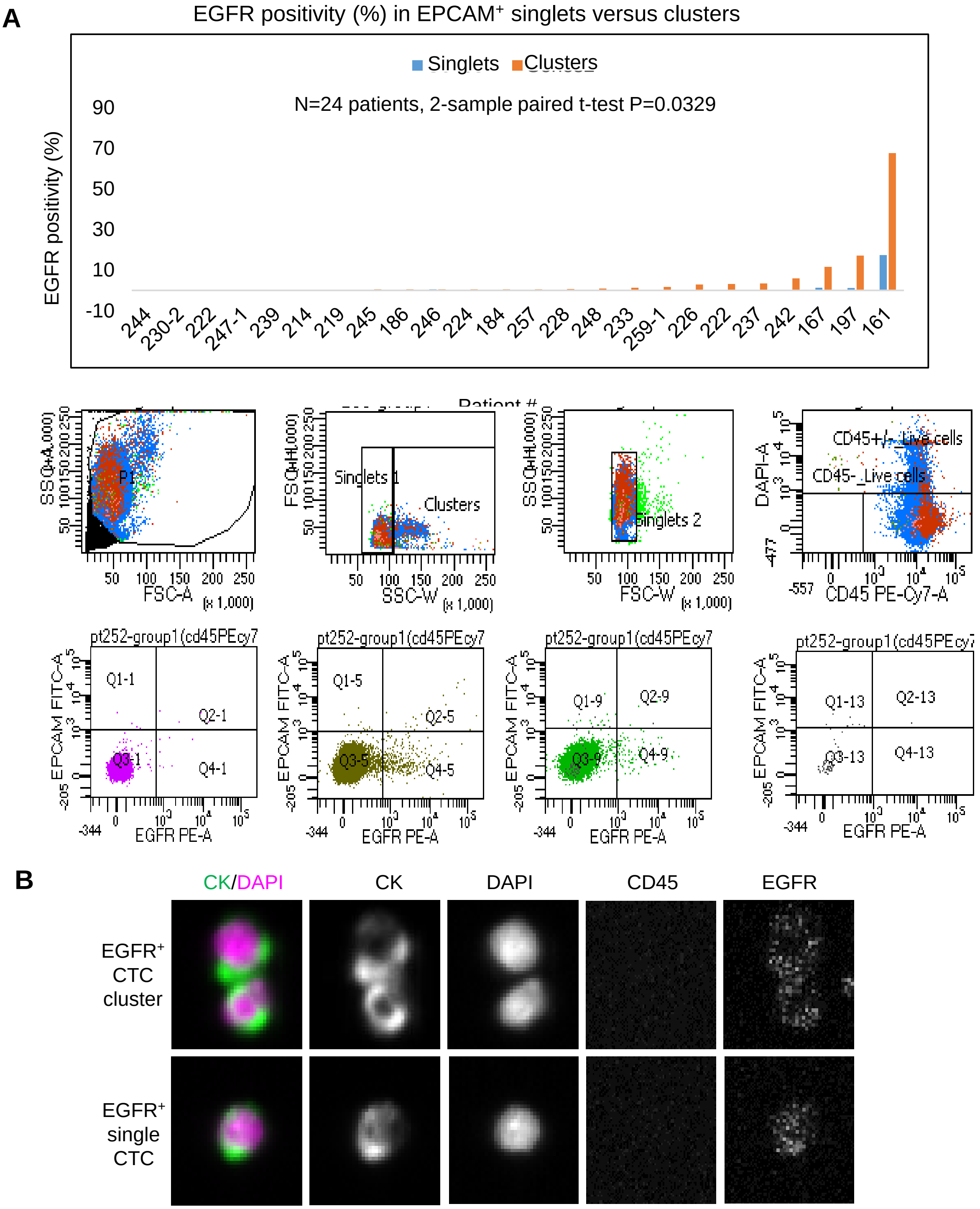

Figure S6. Clustered cells with increased EGFR expression compared to single cells in the blood of breast cancer patients.

A. Top panel: Histogram bars of EGFR expression (%) in single and clustered cells of individual patients with breast cancer, gated from EpCAM+ epithelial cells. Bottom panels: representative flow dot plots of patient blood cells with gated populations of singlets, clusters, DAPI negative live cells, leukocyte marker CD45, and surface protein expression markers CD45 (leukocytes), EpCAM (epithelial cells), and EGFR.

B. Represantative images of EGFR+ CTC clusters and singles aquired from the blood of breast cancer patients, analyzed on CellSearch after EpCAM-binding bead enrichment and immunofluorescence staining with cytokeratin (CK)-FITC, CD45-PE, EGFR-APC and nuclear DNA (DAPI).
